# Internal Layered Reaction Front in 2.5D C/SiC Composites Under Continuous-Wave Laser Ablation: Identification and Thermal-Field Interpretation

**DOI:** 10.3390/ma19112377

**Published:** 2026-06-03

**Authors:** Chuntong Liu, Renke Wang, Yuwei Lv, Yubin Shi

**Affiliations:** 1 College of Missile Engineering, Rocket Force University of Engineering, Xi’an 710025, China; 13772058793@163.com; 2 State Key Laboratory of Laser Interaction with Matter, Northwest Institute of Nuclear Technology, Xi’an 710024, China

**Keywords:** 2.5D C/SiC composite, continuous-wave laser ablation, internal reaction front, surface transition zone, micro-CT, thermal-field simulation

## Abstract

**Highlights:**

- A surface transition zone and a subsurface internal reaction front can be identified in CW laser-ablated 2.5D C/SiC, with the front depth notably exceeding the surface recession depth.
- Combined Micro-CT and SEM/EDS facilitates characterization of subsurface matrix degradation and silicon depletion below the ablated surface.
- The development of the internal reaction front shows a close correlation with simulated through-thickness thermal field, which is likely associated with local thermal history.

**Abstract:**

The ablation behavior of 2.5D C/SiC composites under continuous-wave laser irradiation involves not only surface material removal but also internal structural degradation. In this study, laser ablation tests were conducted at power densities of 400, 800, and 1600 W/cm^2^, and the ablated specimens were analyzed by macroscopic observation, infrared thermography, X-ray micro-computed tomography (micro-CT), cross-sectional scanning electron microscopy/energy-dispersive X-ray spectroscopy (SEM/EDS), depth measurement, and homogeneous thermal-field simulation. The results show that the surface morphology evolved from a transition-zone-dominated response to a typical zoned morphology consisting of a central ablation zone, transition zone, and edge zone as the power density and irradiation time increased. Under the present temperature measurement conditions, the surface transition zone corresponded to an apparent temperature window of approximately 2300–2700 K. Cross-sectional characterization further revealed a distinguishable internal reaction front beneath the external ablation surface, above which microstructural damage and Si depletion were observed. Depth measurements showed that the external ablation depth underestimated the actual degradation depth along the thickness direction. The calibrated homogeneous thermal-field model indicated that the internal front position corresponded to a relatively stable temperature range, suggesting that its formation was mainly governed by local thermal history and matrix-related reactions. The proposed internal reaction front provides a supplementary parameter for evaluating laser-induced subsurface degradation in 2.5D C/SiC composites.

## 1. Introduction

Carbon fiber-reinforced silicon carbide (C/SiC) ceramic matrix composites and carbon/carbon–silicon carbide (C/C–SiC) composites have been widely considered for thermal protection systems, propulsion components, and high-temperature structural applications because of their low density, high specific strength, thermal shock resistance, and oxidation resistance compared with conventional high-temperature materials [1,2,3,4]. Recent studies on hypersonic materials and ceramic matrix composite structures have further emphasized the importance of oxidation resistance, damage tolerance, and thermal-response prediction for composite materials used in extreme aerothermal environments [1,2,3]. In these applications, the materials may be exposed to severe localized thermal loading, where laser irradiation is often used as a controllable method to simulate high-energy thermal ablation and to evaluate the degradation behavior of ceramic matrix composites [5,6,7,8,9,10,11,12,13].

Previous studies have shown that the laser ablation behavior of C/SiC composites is governed by coupled thermophysical and thermochemical processes, including heat conduction, surface recession, SiC oxidation, carbon fiber oxidation, gas-product release, and reaction-product accumulation [5,6,7,8,9,10,11,12,13]. Under high-temperature conditions, SiC may undergo passive oxidation with SiO_2_ formation or active oxidation accompanied by volatile SiO generation, while carbon fibers and pyrolytic carbon interphases are gradually consumed through oxidation reactions [5,8,9,13]. These processes lead to complex surface morphologies, such as central ablation pits, transition regions, oxidation zones, granular products, and exposed fiber skeletons [5,8,9,10,11,12,13]. However, most existing studies mainly evaluate ablation damage using surface morphology, mass loss, linear ablation rate, or surface temperature response.

For 2.5D needle-punched C/SiC composites, the ablation process can be more complex because of the heterogeneous architecture consisting of continuous fiber bundles, chopped-fiber felt layers, Z-direction needling fibers, pores, and SiC matrix [14,15,16,17]. This architecture may cause non-uniform heat transfer, preferential matrix consumption, oxygen penetration, and localized internal damage. Therefore, the degradation of 2.5D C/SiC composites under continuous-wave laser irradiation may not be fully represented by the external ablation surface alone. In particular, a subsurface modified region may form beneath the apparent pit surface before or during obvious geometric material removal.

X-ray micro-computed tomography has been increasingly used to characterize the internal structure, porosity, and damage evolution of fiber-reinforced ceramic matrix composites [18,19]. Combined with cross-sectional SEM and EDS analysis, micro-CT can provide useful evidence for identifying internal density changes, matrix loss, and elemental redistribution after ablation. Meanwhile, infrared thermography and numerical thermal-field modeling can help correlate the observed damage features with the thermal history of the material [18,19]. These methods provide a basis for linking surface ablation morphology, internal structural degradation, and temperature-field evolution.

Despite these advances, the relationship between surface transition-zone formation, subsurface degradation, and internal temperature history in 2.5D C/SiC composites remains insufficiently clarified. In particular, it is still unclear whether the external ablation depth can represent the actual degradation depth along the thickness direction, and whether an identifiable internal reaction front exists beneath the ablated surface. Clarifying this issue is important for evaluating laser-induced damage in 2.5D C/SiC composites, especially when the surface pit does not fully reflect internal matrix-related degradation.

In this study, continuous-wave laser ablation tests were conducted on 2.5D C/SiC composites under different power densities and irradiation times. Macroscopic observation, infrared thermography, micro-CT, cross-sectional SEM/EDS, depth measurement, and homogeneous thermal-field simulation were combined to investigate the surface zonation, internal reaction front, and their correlation with the temperature field. The objectives of this study are: (1) to identify the evolution of surface zonation and the apparent temperature window of the transition zone; (2) to reveal the existence and physical meaning of an internal layered reaction front beneath the external ablation surface; and (3) to interpret the formation of this front based on the internal temperature field and C–Si–O reaction processes. This work provides a supplementary perspective for evaluating subsurface degradation in 2.5D C/SiC composites under continuous-wave laser ablation.

## 2. Materials and Methods

### 2.1. Material Architecture and Specimen Preparation

**Figure 1:**
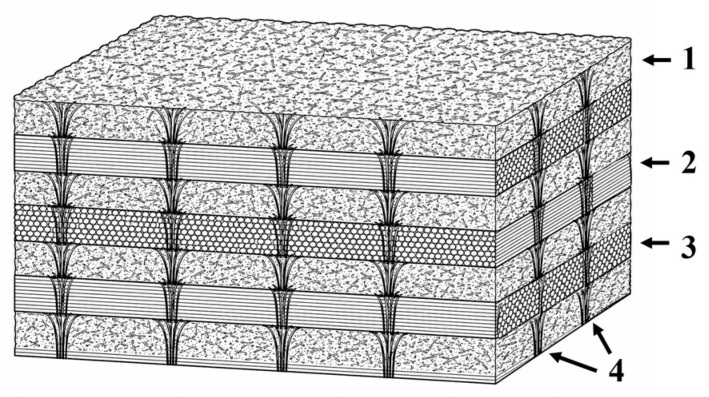
Schematic illustration of the fiber preform architecture of the 2.5D needle-punched C/SiC composite: (1) felt layer; (2) 0° non-woven fiber layer; (3) 90° non-woven fiber layer; and (4) Z-direction needled fibers.

The material used in this study was a 2.5D needle-punched C/SiC ceramic matrix composite supplied by Fanrui Yihui Composite Materials Co., Ltd. (Gongyi, Henan, China). In this work, the X and Y directions denote the in-plane directions of the 0° and 90° non-woven fiber layers, respectively, while the Z direction denotes the through-thickness direction. The fiber preform consisted of alternately stacked 90° non-woven fiber layers, felt layers, and 0° non-woven fiber layers, which were connected through the thickness direction by Z-direction needling fibers. The non-woven fiber layers were mainly composed of continuous carbon fiber bundles and provided the primary in-plane load-bearing framework. The felt layers consisted of randomly oriented chopped carbon fibers, forming a fibrous network with certain pore characteristics. The Z-direction needling fibers penetrated adjacent layers and improved the interlaminar bonding and through-thickness structural continuity, as illustrated in Figure 1. The reinforcement was 12K PAN-based T700 carbon fiber, and the matrix was a SiC ceramic matrix.

Owing to the spatially non-uniform distribution of continuous fiber bundles, chopped-fiber felt layers, Z-direction needling fibers, pores, and the SiC matrix, the composite exhibited typical multi-scale and multi-component heterogeneity [14,15,16,17]. Such structural characteristics may lead to local thermal responses, oxidation reactions, and internal damage evolution that differ from those of homogeneous materials under laser thermal loading. This provides the structural basis for the subsequent analysis of the formation of the internal reaction front.

The specimens were machined into rectangular plates with dimensions of 40 mm × 40 mm × 3 mm. The laser-irradiated side was defined as the front surface, while the opposite side was defined as the back surface. For depth measurement, the positive Z direction was taken from the front surface to the back surface. This direction was selected because surface recession, through-thickness heat transfer, and internal front propagation mainly occurred along the specimen thickness. All external ablation depths and internal front depths were measured using the initial height of the front surface as the reference plane.

### 2.2. Continuous-Wave Laser Ablation and Temperature Measurement

**Figure 2:**
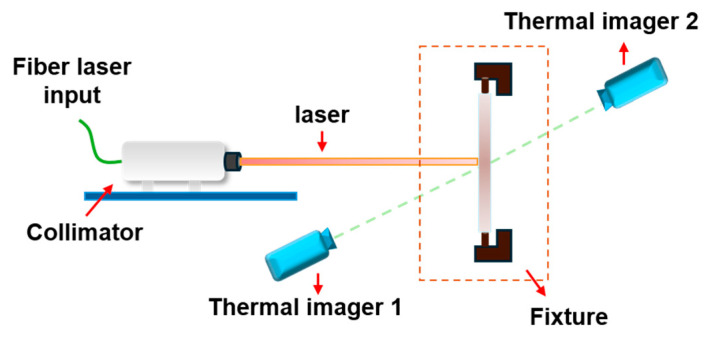
Experimental setup for laser ablation tests.

The continuous-wave laser ablation experimental setup is shown in Figure 2. A fiber laser was used as the heat source, with a laser wavelength of 1070 nm and a spot diameter of 21 mm. After passing through beam-shaping optical elements, the laser beam was normally incident on the front surface of the specimen. The specimen was vertically fixed in a holder, and the temperature responses of the front and back surfaces were synchronously recorded using infrared thermal cameras.

Three laser power densities, namely 400, 800, and 1600 W/cm^2^, were used in this study. For each power density, two irradiation times were selected, giving a total of six experimental conditions. The low-power conditions were used to capture the early thermal response dominated by the transition zone, whereas the medium- and high-power conditions were used to obtain distinct surface pits and internal reaction fronts. The detailed experimental conditions are listed in Table 1.

The front- and back-surface temperatures were synchronously recorded using FLIR infrared thermal cameras. The frame rate was 25 Hz, the spatial resolution was 640 × 480 pixels, the emissivity was set to 0.95, and the temperature acquisition range was 500–3300 K. In this study, the apparent temperature data exported from the thermal camera software (FLIR Tools 6.4) were used for analysis without additional emissivity correction [18,19]. For local regions where the temperature approached or exceeded the calibrated measurement range of the instrument, the corresponding data were used only to identify the temperature evolution trend and the location of the high-temperature region, rather than as precise absolute temperatures.

### 2.3. Post-Ablation Characterization and Depth Measurement

**Figure 3:**
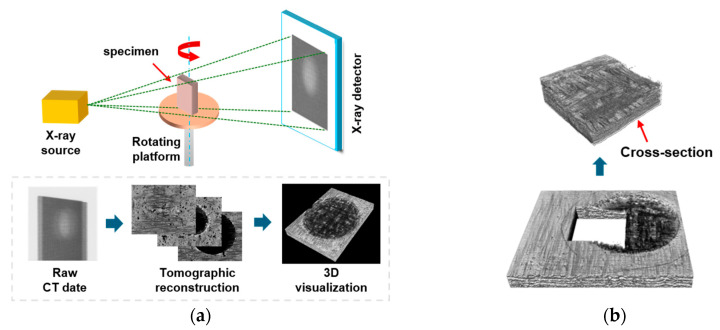
Schematic illustration of micro-CT reconstruction and central-section preparation after laser ablation. (**a**) Micro-CT scanning and reconstruction; (**b**) central-section preparation for SEM/EDS.

After laser ablation, the surface morphologies of the specimens were recorded using a digital camera and a stereomicroscope. Based on surface color, roughness, product distribution, and geometric removal characteristics, the ablated surface was divided into a central ablation zone, a transition zone, and an edge zone. The micro-CT reconstruction and central-section preparation procedures are shown in Figure 3.

X-ray micro-CT was used to examine the internal structure and center cross-sectional morphology of the ablated specimens [20,21,22,23]. The scanning was performed using a Bruker SkyScan 1275 system (Bruker Belgium, Kontich, Belgium). The scanning voltage, current, exposure time, filter, rotation step, and voxel size were 70 kV, 80 μA, 100 ms, 1 mm Al, 0.5°, and 25 μm, respectively. To ensure comparability among different specimens, all CT data were reconstructed using consistent parameters, with the Reference Intensity set to 58,000.

The external ablation depth and internal front depth were measured from CT cross-sections passing through the laser-spot center. The unaffected surrounding front surface was used as the initial reference plane. The external ablation depth was defined as the recession depth of the central geometric surface relative to the initial front surface. The internal front depth was defined as the distance from the initial front surface to the internal state-transition boundary identified from CT grayscale contrast. The difference between these two depths was used to estimate the thickness of the modified region beneath the external ablation surface.

To further verify the physical meaning of the CT-identified internal boundary, a representative specimen was sectioned along the laser centerline, as schematically shown in Figure 3b. The cross-section was ground and polished before SEM/EDS characterization. SEM observations and EDS elemental mapping were performed using a Zeiss Sigma 300 (Carl Zeiss AG, Oberkocken, Germany) scanning electron microscope equipped with an energy-dispersive X-ray spectroscopy system. SEM was used to observe the cross-sectional microstructural damage, while EDS was used to analyze the distributions of C, Si, and O across the modified region and the relatively intact region. The accelerating voltage was adjusted within 3–10 kV according to the observation magnification and analysis requirement. The SEM/EDS observations were conducted under comparable imaging and mapping conditions for the modified region and the relatively intact region to ensure the consistency of elemental-distribution comparison.

### 2.4. Homogeneous Thermal-Field Model

#### 2.4.1. Model Assumptions

To relate the experimentally identified internal reaction front to the internal temperature field, a homogeneous thermal-field model was established. The 2.5D C/SiC composite was treated as an equivalent continuous medium, without explicitly resolving fiber bundles, felt layers, pores, and interfacial phases [16,24,25,26,27]. This treatment was adopted to describe the overall thermal response of the specimen rather than the local fiber-scale morphology or pore evolution. The temperature-dependent thermal conductivity and specific heat capacity used in the model were based on the effective properties of the initial composite. Porosity evolution, density reduction, and degradation-induced changes in the local thermophysical properties of the low-density modified layer were not explicitly coupled in the present model. Therefore, the model should be regarded as a simplified thermal-field interpretation rather than a full thermal-chemical-structural degradation model.

The numerical model was implemented in COMSOL Multiphysics 6.4. The model geometry was established according to the experimental specimen dimensions of 40 mm × 40 mm × 3 mm, and the laser spot diameter, power density, and irradiation duration were set consistently with the experimental conditions listed in Table 1. The model was used to calculate the transient through-thickness temperature field and to provide a semi-quantitative interpretation of the experimentally identified internal reaction front, rather than to reproduce the local heterogeneous morphology of the real composite.

The model considered transient heat conduction, Gaussian laser heat-flux input, convective and radiative heat losses, and equivalent high-temperature surface recession. The thermal conductivity and specific heat capacity were defined as temperature-dependent functions. The absorptivity, equivalent heat-loss term, and recession parameters were constrained by the measured temperature response and external ablation depth, so that the model could reproduce the main thermal-ablation trends observed in the experiments.

#### 2.4.2. Governing Equation and Boundary Conditions

The transient temperature field was calculated using the heat conduction equation:

(1) $$\rho C_{p}(T)\frac{\partial T}{\partial t}=\nabla \cdot [k(T)\nabla T]$$

where ρ is the equivalent density, $C_{p}(T)$ is the temperature-dependent specific heat capacity, k(T) is the temperature-dependent equivalent thermal conductivity, T is the temperature, and t is the time.

The laser beam was applied to the front surface as a circular Gaussian heat-flux boundary:

(2) $$q_{laser}(r,t)=\eta q_{0}exp[-2{\left(\frac{r}{r_{0}}\right)}^{2}]f(t)$$

where $q_{laser}$ is the absorbed laser heat flux, η is the surface absorptivity, $q_{0}$ is the central heat flux, r is the radial distance from the beam center, $r_{0}$ is the laser spot radius, and f(t) is the temporal loading function. A smoothed step function was used at the beginning of laser loading to improve numerical stability, while the total loading time was kept consistent with the experiment.

On the laser-irradiated surface, the thermal boundary condition included laser input, convective heat loss, radiative heat loss, and an equivalent ablation-related heat-loss term:

(3) $$-n\cdot [-k(T)\nabla T]=q_{laser}-h(T_{s}-T_{\infty })-\varepsilon \sigma (T_{s}^{4}-T_{\infty }^{4})-Q_{ab}$$

where n is the outward normal vector, $T_{s}$ is the local surface temperature, $T_{\infty }$ is the ambient temperature, h is the convective heat transfer coefficient, ε is the surface emissivity, σ is the Stefan–Boltzmann constant, and $Q_{ab}$ is the equivalent heat-loss term associated with high-temperature ablation, oxidation, decomposition, and volatilization [24,25,26,27]. The other non-irradiated surfaces were subjected only to convective and radiative heat exchange with the ambient environment.

The main user-defined parameters are listed in Table 2 [24,25,26,27]. The temperature-dependent thermal conductivity and specific heat capacity are provided in Supplementary Tables S1 and S2.

#### 2.4.3. Equivalent Surface Recession

When the front-surface temperature reached the high-temperature ablation range, a moving mesh method was used to describe the normal recession of the irradiated surface [24,25,27]. The recession velocity was defined as:

(4) $$v_{n}=v_{\mathrm{rec}}(T_{s})$$

where $v_{n}$ is the normal mesh velocity and $v_{\mathrm{rec}}(T_{s})$ is the temperature-dependent equivalent recession velocity. The recession direction was set from the initial front surface toward the interior of the specimen.

The recession velocity used here is an equivalent boundary parameter rather than the intrinsic rate of a single reaction process. It was determined from the experimental external ablation depth and temperature response, and was used to update the surface position and thermal boundary during laser irradiation. Therefore, the surface recession module served mainly to improve the internal temperature-field calculation, rather than to independently predict the real local ablation morphology. The detailed recession-velocity function is given in Supplementary Table S3. For all simulated conditions, the same material-property functions, heat-transfer boundary settings, and recession-velocity function were used. Only the laser power density and irradiation duration were changed according to Table 1, so that the calculated temperature fields could be compared under consistent modeling assumptions.

## 3. Results and Discussion

### 3.1. Evolution of Surface Zonation

**Figure 4:**
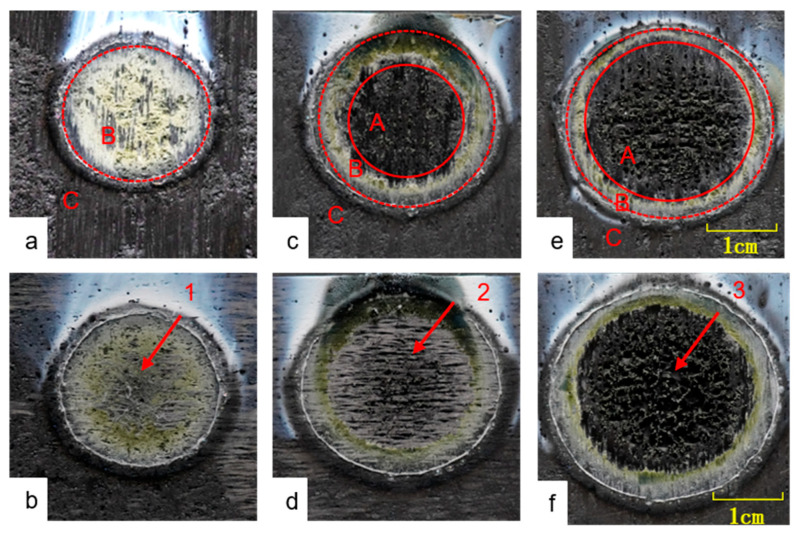
Evolution of surface morphologies of 2.5D C/SiC under different laser power densities and irradiation times. A: central ablation zone; B: transition zone; C: edge zone. 1: flat center; 2: slightly depressed center; 3: severely depressed center. (**a**) 400 W/cm^2^, 6 s; (**b**) 400 W/cm^2^, 12 s; (**c**) 800 W/cm^2^, 6 s; (**d**) 800 W/cm^2^, 12 s; (**e**) 1600 W/cm^2^, 3 s; (**f**) 1600 W/cm^2^, 6 s.

Figure 4 shows the surface morphologies of the 2.5D C/SiC composites after continuous-wave laser ablation under different power densities and irradiation times. With increasing power density, the surface response evolved from local thermal modification to distinct pit ablation. According to the central geometric removal, surface color, roughness, and annular product distribution, the ablated surface can be divided into a central ablation zone, a transition zone, and an edge zone, denoted as A, B, and C, respectively [5,8,9,10,12]. The central ablation zone corresponds to the region with the strongest laser energy input and the most severe material removal. The edge zone mainly shows weak thermal influence or slight oxidation. The transition zone is located between these two regions and is characterized by annular distribution, color contrast, roughened morphology, and local product accumulation [5,8,9,13].

At 400 W/cm^2^, no distinct central pit was formed. The ablation center remained relatively flat, while an annular modified region appeared within the irradiated area. This indicates that the surface had undergone thermal oxidation, matrix-related reactions, or reaction-product formation before significant geometric removal occurred. Therefore, the 400 W/cm^2^ condition represents an early stage dominated by surface reaction and near-surface modification.

At 800 W/cm^2^, the ablation center changed from a flat surface to a slightly depressed region, accompanied by more obvious roughening and material removal. The transition zone appeared as an annular region outside the central pit. At 1600 W/cm^2^, the central depression became more pronounced, the pit boundary became clearer, and the transition zone was located between the strongly ablated center and the weakly affected peripheral region. These results show that the transition zone is a thermally responsive region associated with local temperature attenuation and reaction-product distribution, rather than merely a visual boundary formed after pit development.

**Figure 5:**
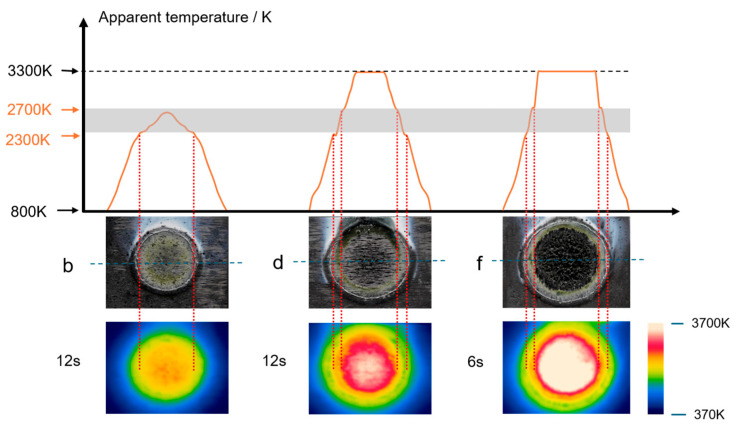
Correlation between the surface transition-zone boundaries and the infrared temperature field. Conditions b, d, and f correspond to 400 W/cm^2^—12 s, 800 W/cm^2^—12 s, and 1600 W/cm^2^—6 s, respectively. Under the present temperature measurement conditions, the apparent temperature window of the transition zone is approximately 2300–2700 K.

To correlate surface zonation with the temperature field, the transition-zone boundaries identified from the post-ablation morphology were compared with the front-surface infrared temperature field at the end of laser irradiation, as shown in Figure 5. All six conditions were examined. Conditions a, c, and e represent the shorter irradiation-time cases at 400, 800, and 1600 W/cm^2^, respectively, and were used to observe the early-stage development of surface zonation under different power densities. In these cases, the transition-zone boundaries were relatively weak, incomplete, or more strongly affected by transient surface evolution, which increased the uncertainty of quantitative boundary-temperature extraction. Therefore, conditions b, d, and f, corresponding to the longer irradiation-time cases under the same three power densities, were selected to estimate the apparent temperature window of the transition zone. In these cases, the transition zone was more fully developed, and its inner and outer boundaries were clearer and more continuous. Under the present infrared measurement and data-processing conditions, the outer and inner boundaries of the transition zone mainly corresponded to approximately 2300 K and 2700 K, respectively. Thus, 2300–2700 K can be regarded as an empirical apparent temperature window for transition-zone formation in this study.

It should be noted that the central high-temperature regions under 800 and 1600 W/cm^2^ were displayed as 3300 K because the temperature approached or exceeded the calibrated measurement range. This treatment mainly affected the display of the central plateau and did not change the determination of the transition-zone boundary temperatures. In addition, the infrared temperature data were affected by emissivity setting, surface morphology, reaction products, and high-temperature radiation conditions. Therefore, the 2300–2700 K range should not be interpreted as an absolute reaction threshold, but as an empirical criterion linking surface morphology and temperature field under the same measurement conditions [18,19].

This temperature window helps explain the surface morphological evolution. At 400 W/cm^2^, the front-surface temperature was mainly close to or partly within the 2300–2700 K range, so the surface response was dominated by transition-zone reactions and near-surface modification. With increasing power density, the center temperature exceeded this window and entered a stronger material-removal regime, while the transition zone shifted outward to the annular region where the temperature decayed to 2300–2700 K. Therefore, surface zonation reflects not only the spatial difference in ablation severity, but also the temperature-field distribution that governs the formation of the transition zone.

### 3.2. Evidence for the Internal Layered Reaction Front

**Figure 6:**
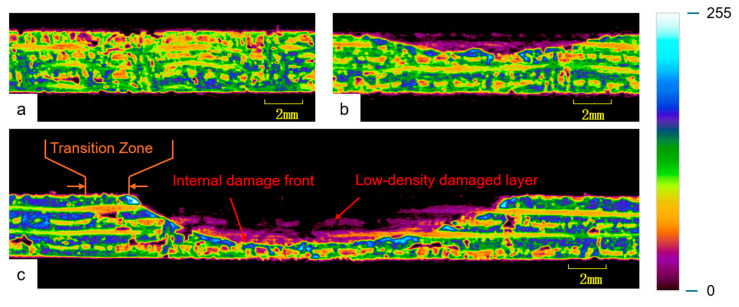
Central cross-sectional micro-CT images of 2.5D C/SiC specimens after ablation: (**a**) 400 W/cm^2^ for 12 s; (**b**) 800 W/cm^2^ for 12 s; and (**c**) 1600 W/cm^2^ for 6 s.

Although surface morphology reflects the external ablation state, it cannot fully describe the degradation extent along the thickness direction of the composite. Figure 6 shows the micro-CT center cross-sections of the ablated specimens under different power densities. The pseudo-color display was used to enhance the CT grayscale contrast; the color variation reflects differences in X-ray attenuation and apparent density, rather than direct chemical composition [20,21,22,23].

With increasing power density, the internal structure evolved from slight near-surface modification to a layered damaged region with a distinguishable boundary. At 400 W/cm^2^, the cross-section maintained good structural continuity, and only limited low-grayscale regions were observed near the irradiated surface. Neither obvious surface recession nor a continuous internal boundary was formed, indicating that the damage was mainly restricted to near-surface modification. At 800 W/cm^2^, a continuous low-density region appeared beneath the surface, and an approximately arc-shaped internal boundary was formed, although the external surface recession remained limited. At 1600 W/cm^2^, the central pit became more pronounced, and a thicker low-density modified layer developed beneath the external ablation surface. The boundary between this modified layer and the relatively intact region was also clearer.

In the 1600 W/cm^2^ specimen, the surface transition zone showed spatial continuity with the internal boundary in the CT cross-section. This suggests that the surface transition zone and the internal low-density layer are related manifestations of the same thermally driven degradation process. The former reflects surface reaction and product accumulation, whereas the latter records the subsurface advancement of matrix-related damage.

**Figure 7:**
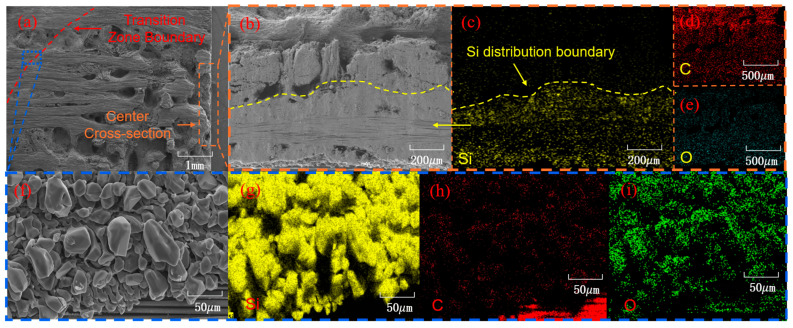
Cross-sectional SEM/EDS evidence of the internal reaction front and transition-zone products under 1600 W/cm^2^ for 6 s: (**a**) Low-magnification surface SEM image of the ablated region; (**b**) cross-sectional SEM image showing the modified layer and internal reaction front; (**c**–**e**) cross-sectional EDS elemental maps of C, Si, and O, respectively; (**f**) high-magnification SEM image of the transition zone; and (**g**–**i**) EDS elemental maps of Si, C, and O in the transition zone, respectively.

The physical meaning of this internal boundary was further examined by cross-sectional SEM and EDS, as shown in Figure 7. The SEM image reveals obvious structural loosening and matrix loss in the region between the external ablation surface and the internal boundary, while the fiber/matrix structure below this region remained relatively continuous. The EDS mapping shows a clear transition in the Si distribution, which is generally consistent with the structural boundary observed in SEM and CT. In contrast, the C and O distributions were less sharply bounded. Since Si mainly originates from the SiC matrix, the rapid decrease in the Si signal near the internal front can be attributed to matrix-related oxidation, volatilization, and redistribution. Under relatively oxygen-rich conditions, SiC may undergo passive oxidation and form SiO_2_, whereas under high-temperature and locally oxygen-deficient conditions, active oxidation may generate volatile SiO and CO species [5,13,28]. These reactions promote the consumption or loss of the Si-containing matrix near the front, leading to a clearer Si distribution boundary. By contrast, the C and O signals are less distinct because carbon fibers, residual carbon, oxide products, and local pores may coexist within the thermally modified transition region.

High-magnification SEM/EDS results near the transition zone further show nodular or crystalline product accumulation with a relatively strong Si signal, whereas the exposed fiber region in the central ablation zone exhibited a much weaker Si signal. This confirms that the transition zone is not only a macroscopic color or roughness boundary, but also a region associated with residual or accumulated Si-containing reaction products [5,8,9,13].

Based on the CT, SEM, and EDS evidence, the internal boundary is defined here as an internal layered reaction front. Here, the term “front” does not imply an ideal flat interface or an abrupt chemical boundary. Instead, it refers to a material state-transition region identifiable from CT grayscale contrast, cross-sectional morphology, and Si distribution. Due to the heterogeneous architecture of the 2.5D C/SiC composite, local pores, fiber-bundle orientation, and needling-induced structural heterogeneity may locally affect heat transfer, gas diffusion, and reaction pathways, leading to a wavy or diffuse front morphology at the microscopic scale. Therefore, this front represents an apparent and characteristic boundary between the severely thermally modified region and the relatively intact region, and provides a useful position for evaluating subsurface degradation in 2.5D C/SiC composites.

The external ablation depth and internal front depth were measured from the center cross-sections, and the results are summarized in Table 3. Under 400 W/cm^2^, neither external recession nor a continuous internal front was quantitatively identifiable. At 800 W/cm^2^, the external ablation depth increased slightly from 0.19 mm at 6 s to 0.25 mm at 12 s, while the internal front depth remained much larger, increasing from 1.05 mm to 1.10 mm. Under 1600 W/cm^2^, both the external ablation depth and the internal front depth increased markedly. At 3 s, the external ablation depth was 0.63 mm, whereas the internal front depth reached 1.74 mm. At 6 s, these values increased to 1.17 mm and 2.09 mm, respectively.

For all identifiable conditions, the internal front depth was approximately 0.85–1.11 mm greater than the external ablation depth. This difference demonstrates that the surface pit depth alone underestimates the actual degradation depth along the thickness direction. Therefore, the internal reaction front provides a more representative parameter for describing laser-induced subsurface damage in 2.5D C/SiC composites.

### 3.3. Model Evaluation and Internal Temperature Field Distribution

After the internal reaction front was experimentally identified, the homogeneous thermal-field model was used to examine its relationship with the internal temperature distribution. As described in Section 2.4, the 2.5D C/SiC composite was treated as an equivalent continuous medium rather than explicitly resolving fiber bundles, felt layers, pores, and interfacial phases [16,24,25,26,27]. Therefore, the model was not intended to reproduce the local heterogeneous ablation morphology, but was used as a semi-quantitative tool to obtain the overall thermal response associated with the experimentally observed surface recession and internal front depth. Accordingly, the simulated temperature field was used to interpret the thermal history associated with the internal front position, while the possible decrease in thermal conductivity caused by porosity and matrix loss in the degraded layer was treated as a limitation of the present model.

**Figure 8:**
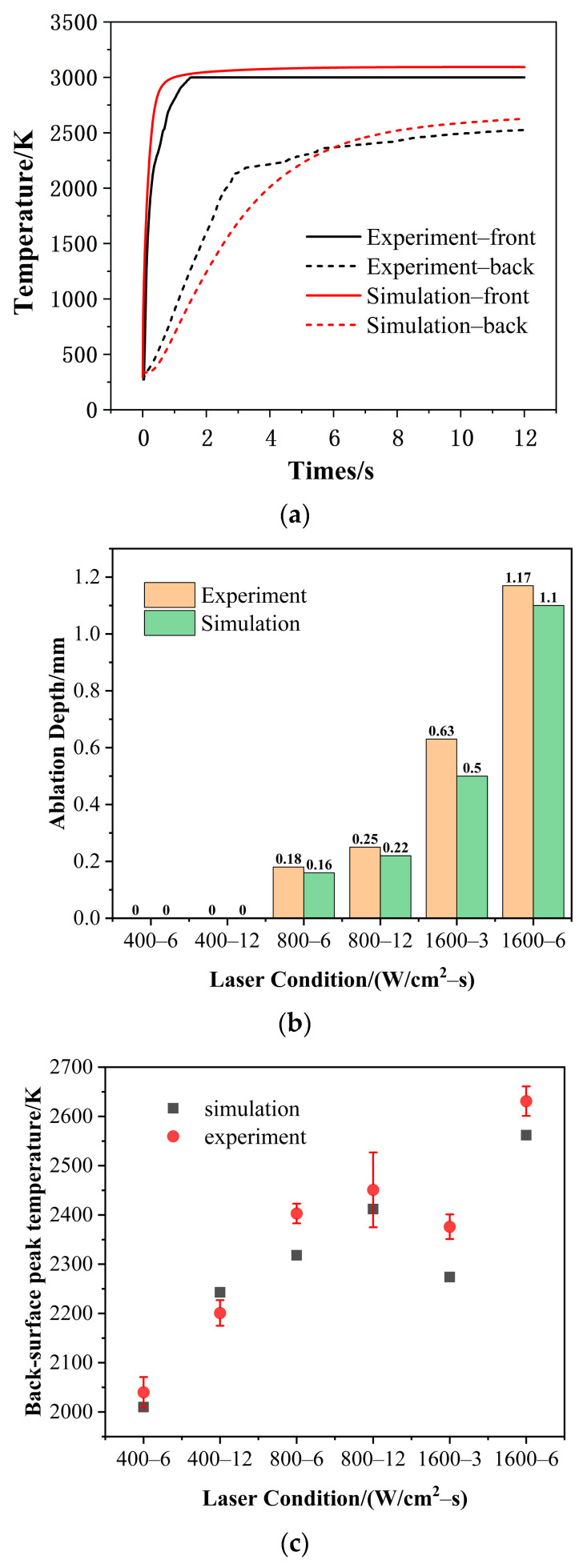
Comparison between experimental and simulated thermal response and ablation behavior: (**a**) front- and back-surface temperature histories at 400 W/cm^2^ for 12 s; (**b**) comparison of experimental and simulated external ablation depth; and (**c**) comparison of experimental and simulated back-surface peak temperature.

Figure 8 compares the experimental and simulated thermal response and external ablation behavior. As shown in Figure 8a, under the 400 W/cm^2^, 12 s condition, the simulation reproduced the main features of the measured temperature histories. The front surface rapidly reached a high-temperature plateau, whereas the back surface exhibited a delayed and more gradual temperature rise. The difference between the experimental and simulated peak back-surface temperatures was approximately 100 K, indicating that the model captured the main through-thickness heat-transfer behavior.

The calculated external ablation depth was further compared with the experimental measurement, as shown in Figure 8b. The model reproduced the overall trend of enhanced surface recession with increasing power density and irradiation time. Under 400 W/cm^2^, neither the experiment nor the simulation showed obvious surface recession. Under 800 W/cm^2^, the calculated recession depth remained small and was close to the experimental value. Under 1600 W/cm^2^, both the experimental and simulated results showed a marked increase in surface recession. In addition, Figure 8c shows that the simulated back-surface peak temperatures were generally consistent with the experimental values under different conditions. The remaining deviations may be associated with the heterogeneous distribution of fiber bundles, felt layers, pores, residual carbon fibers, surface emissivity variation, and heat loss through the specimen holder [14,15,16,17,18,19,24,25,26,27]. Nevertheless, the agreement in both external ablation depth and back-surface peak temperature provides a reasonable basis for using the model to analyze the internal temperature field.

**Figure 9:**
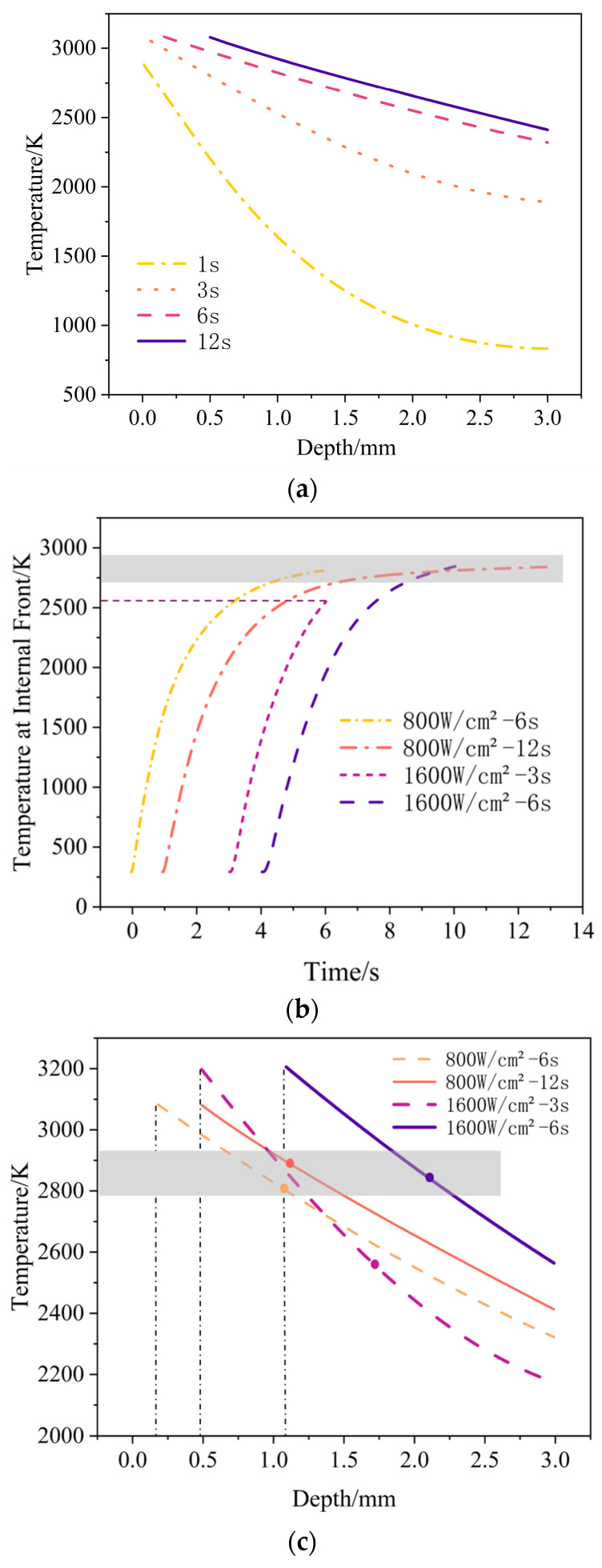
Simulated internal temperature field and its relation to the experimentally observed internal front. (**a**) Evolution of axial temperature profiles at 800 W/cm^2^. (**b**) Temperature histories extracted at the experimentally measured internal front depths. (**c**) Final axial temperature profiles under different conditions, with solid dots indicating the experimentally measured internal front positions.

Based on the above model evaluation, the axial temperature distribution along the ablation centerline was further analyzed. Figure 9a shows four representative centerline axial temperature profiles under the 800 W/cm^2^ condition. At the early stage of laser loading, the temperature was concentrated near the irradiated surface, and a large axial temperature gradient was formed. As irradiation continued, heat gradually transferred into the interior, and the temperature profile evolved from a strongly nonlinear distribution to a smoother, nearly linear distribution before laser shutdown. This indicates that, under longer irradiation times, the internal thermal field approached a quasi-steady conduction state, making the final axial temperature distribution useful for interpreting the experimentally observed internal front position.

To reduce the influence of model surface-recession error on the analysis of the internal front, the experimentally measured internal front depth was used as the sampling coordinate. The temperature histories at these depths were then extracted from the simulated temperature field, as shown in Figure 9b. For clarity, the curves in Figure 9b are visually offset to reduce overlap, while the temperature values used for analysis were taken from the original simulated data. Under most 800 and 1600 W/cm^2^ conditions, the temperatures at the internal front positions increased with irradiation time and finally concentrated within approximately 2700–2900 K. This result suggests that the internal reaction front was not directly determined by the external pit depth, but was more closely related to the local thermal exposure experienced inside the material.

Figure 9c further compares the final axial temperature profiles under different conditions. The left endpoint of each profile corresponds to the external ablation surface after surface recession, and the projected guide lines indicate the corresponding external ablation depths on the depth axis. Solid dots mark the experimentally measured internal front positions. The internal fronts were generally located deeper than the external ablation surfaces and corresponded to regions where the simulated temperature had reached a high-temperature interval. This correspondence supports the interpretation that the CT-identified internal boundary represents a temperature-history-related reaction front inside the material.

An exception was observed under the 1600 W/cm^2^, 3 s condition, where the simulated final temperature at the internal front position was lower than those under the other conditions. This difference may be related to the strong transient effect under short-duration high-power loading. In this case, rapid surface heating, intense surface recession, heterogeneous heat transfer, and uncertainty in CT-based front identification may all increase the deviation between the equivalent model and the real material response. Therefore, the temperature range obtained from the model should not be interpreted as a fixed critical temperature for front formation, but rather as an apparent temperature interval under the present experimental and modeling conditions.

The simulated axial temperature fields also explain why the external ablation surface and the internal reaction front did not coincide. The external ablation surface was mainly governed by high-temperature material removal and geometric recession at the irradiated surface, whereas the internal front corresponded to the depth at which matrix loss, reconstruction, or Si depletion became identifiable after heat transfer into the material. Thus, the combined experimental and modeling results indicate that continuous-wave laser ablation of 2.5D C/SiC composites involves not only surface recession, but also the advancement of a temperature-history-related internal reaction front.

### 3.4. Coupling Mechanism of Surface Transition and Internal Front Formation

The above results suggest that continuous-wave laser ablation of 2.5D C/SiC composites is not a simple surface-by-surface removal process. Instead, surface zoned reactions, external surface recession, and subsurface front advancement occur simultaneously. This coupled response can be interpreted by considering the high-temperature reactions of the SiC matrix, carbon fibers, and gaseous products in the C–Si–O system.

In high-temperature oxidation and ablation environments, the degradation of C/SiC composites is mainly governed by SiC matrix oxidation, carbon oxidation, volatilization of reaction products, and local thermal decomposition [5,6,7,28,29,30,31]. Depending on temperature and oxygen partial pressure, SiC oxidation may proceed through passive oxidation, which forms a SiO_2_ layer, or active oxidation, which generates volatile SiO and leads to continuous SiC consumption [28,29,30,31]. The representative reactions can be expressed as follows:

(5) $$SiC(s)+\frac{3}{2}O_{2}(g)\to SiO_{2}(s/l)+CO(g)$$

(6) $$SiC(s)+O_{2}(g)\to SiO(g)+CO(g)$$

For carbon fibers and the pyrolytic carbon interphase, oxidation mainly produces CO and CO_2_ [5,6,17]:

(7) $$C(s)+O_{2}(g)\to CO_{2}(g)$$

(8) $$2C(s)+O_{2}(g)\to 2CO(g)$$

(9) $$C(s)+CO_{2}(g)\to 2CO(g)$$

During laser ablation, these reactions occur in a strongly non-uniform temperature field. In the transition zone, where the apparent temperature was approximately 2300–2700 K under the present measurement conditions, SiC oxidation and the retention of Si-containing reaction products were more evident than severe geometric removal. This explains the annular morphology, color contrast, and nodular or crystalline product accumulation observed in this region. The Si-rich products detected by SEM/EDS near the transition zone further indicate that this zone is not only a visual boundary, but also a reaction band associated with residual, accumulated, or locally redistributed SiC matrix products [5,8,9,13,28,29,30].

By contrast, the central ablation zone experienced a higher heat input and more intense material removal. In this region, the volatilization of SiO, the release of CO and CO_2_, and the rupture or removal of SiO_2_ weakened the protective effect of oxidation products. Once the SiC matrix was consumed or removed, carbon fibers were more directly exposed to the high-temperature oxidative environment, resulting in surface roughening and pit formation [5,6,7,13,28,29,30,31]. Therefore, the transition from the surface transition zone to the central ablation zone reflects a change from reaction-product formation and retention to high-temperature material removal.

The formation of the internal reaction front can be regarded as the extension of this thermochemical response into the thickness direction. The CT results revealed a low-density modified layer beneath the external ablation surface, while SEM/EDS confirmed Si depletion and a relatively clear Si distribution boundary in the same region. Since Si mainly originates from the SiC matrix, this boundary indicates a transition from a matrix-consumed or matrix-reconstructed region to a relatively intact region. Its formation may be attributed to three coupled factors: heat conduction along the thickness direction, oxygen and gaseous-product transport through pores, interlayer channels, and fiber-bundle interfaces, and preferential consumption or volatilization of the SiC matrix in the thermally affected region [14,15,16,17,20,21,22,23].

The modeling results further support this interpretation. The experimentally measured internal front positions were not directly determined by the external pit depth, but corresponded to a relatively concentrated internal temperature range under most 800 and 1600 W/cm^2^ conditions. This indicates that the internal front is more closely related to local thermal history than to surface recession alone. When a certain internal depth experiences sufficient thermal exposure, matrix-related reactions and structural loosening become identifiable by CT, SEM, and EDS, forming the internal layered reaction front.

It should be emphasized that this front should not be regarded as an ideal reaction interface with a fixed critical temperature. The lower simulated temperature at the front position under the 1600 W/cm^2^—3 s condition indicates that short-duration high-power loading involves stronger transient effects, rapid surface recession, and greater sensitivity to structural heterogeneity. Therefore, the internal front is better understood as an apparent state-transition boundary controlled by both temperature history and the heterogeneous architecture of the 2.5D C/SiC composite.

Overall, the ablation behavior of 2.5D C/SiC composites under continuous-wave laser irradiation can be described as a coupled surface–subsurface degradation process. The external ablation surface reflects geometric material removal, the transition zone reflects the formation and retention of surface reaction products, and the internal reaction front reflects the depth of matrix-related thermal degradation. Thus, using only the surface pit depth to evaluate ablation damage would underestimate the actual degradation extent along the thickness direction.

## 4. Conclusions

This study investigated the surface zonation, internal reaction front, and temperature-field correlation of 2.5D C/SiC composites under continuous-wave laser ablation. The main conclusions are as follows.

1. With increasing laser power density and irradiation time, the surface morphology evolved from a transition-zone-dominated state to a typical zoned structure consisting of a central ablation zone, transition zone, and edge zone. Under the present measurement conditions, the transition zone corresponded to an apparent temperature window of approximately 2300–2700 K, indicating that it reflects an early thermo-chemical response rather than only a morphological feature.
2. A distinguishable internal reaction front was identified beneath the external ablation surface. Cross-sectional characterization showed that the region above this front exhibited microstructural damage and Si depletion, whereas the region below it remained relatively intact. Depth measurements further showed that the external ablation depth underestimated the actual degradation depth, especially under high-power conditions.
3. The calibrated homogeneous thermal-field model reasonably reproduced the main trends of back-surface temperature and external ablation depth, and provided a semi-quantitative explanation for the internal front position. The internal reaction front was associated with a relatively stable temperature range in the simulated field, suggesting that its formation was mainly controlled by local thermal history and matrix-related reactions. Therefore, this front can serve as a supplementary characteristic parameter for evaluating laser-induced subsurface degradation in 2.5D C/SiC composites.

## Figures and Tables

**Table 1:** Experimental conditions for continuous-wave laser ablation tests.

Condition	Power Density/W·cm^−2^	Irradiation Time/s
a	400	6
b	400	12
c	800	6
d	800	12
e	1600	3
f	1600	6

**Table 2:** Main thermophysical parameters used in the homogeneous thermal-field model [24,25,26,27].

Parameter	Symbol	Value	Unit
Equivalent density	ρ	2100	kg·m^−3^
Surface emissivity	ε	0.95	-
Ambient temperature	$T_{\infty }$	293.15	K
Initial temperature	$T_{0}$	293.15	K
Convective heat transfer coefficient	h	15	W·m^−2^·K^−1^

**Table 3:** Quantitative comparison between external ablation depth and internal front depth. The condition labels a–f correspond to the experimental conditions listed in Table 1.

Condition Label	Condition	External Ablation Depth/ mm	Internal Front Depth/ mm	Difference/ mm
a	400 W/cm^2^, 6 s	Not identifiable	Not identifiable	-
b	400 W/cm^2^, 12 s	Not identifiable	Not identifiable	-
c	800 W/cm^2^, 6 s	0.19	1.05	0.86
d	800 W/cm^2^, 12 s	0.25	1.10	0.85
e	1600 W/cm^2^, 3 s	0.63	1.74	1.11
f	1600 W/cm^2^, 6 s	1.17	2.09	0.92

## Data Availability

The original contributions presented in this study are included in the article/Supplementary Material. Further inquiries can be directed to the corresponding author.

## Supplementary Materials

The following supporting information can be downloaded at: https://www.mdpi.com/article/10.3390/ma19112377/s1, Table S1: Temperature-dependent specific heat capacity used in the homogeneous thermal-field model; Table S2: Temperature-dependent thermal conductivity used in the homogeneous thermal-field model; Table S3: Temperature-dependent equivalent recession velocity and auxiliary heat-loss terms.

## Abbreviations

The following abbreviations are used in this manuscript:

C/SiC	carbon fiber-reinforced silicon carbide
C/C–SiC	carbon/carbon–silicon carbide
CW	continuous-wave
micro-CT	X-ray micro-computed tomography
SEM	scanning electron microscopy
EDS	energy-dispersive X-ray spectroscopy
PAN	polyacrylonitrile
